# Breastfeeding practices in nursing mothers during COVID-19 pandemic: Dual center study in Karachi, Pakistan

**DOI:** 10.12669/pjms.39.2.6707

**Published:** 2023

**Authors:** Shireen Qassim Bham, Khalid Mahmood Ahmad Khan, Sagheera Anjum Munaver, Umer Hayat Ahmed Sharif

**Affiliations:** 1 Shireen Qassim Bham, Associate Professor, Department of Pediatrics, Fazaia Ruth Pfau Medical College, Karachi, Pakistan; 2 Khalid Mahmood Ahmad Khan, Professor of Pediatrics, Fazaia Ruth Pfau Medical College, Karachi, Pakistan; 3 Sagheera Anjum Munaver, Associate Professor, Department of OBGYN, Fazaia Ruth Pfau Medical College, Karachi, Pakistan; 4 Umer Hayat Ahmed Sharif, Medical student, Dow University of Health Sciences, Karachi, Pakistan

**Keywords:** COVID-19 Pandemic, Breast Feeding Practices, Nursing mothers, Child health

## Abstract

**Objective:**

To identify the breastfeeding practices in nursing mothers during the COVID-19 pandemic.

**Methods:**

This descriptive cross-sectional study was conducted in the Pediatrics and Gynecology & Obstetrics departments at two of the tertiary care hospitals in Pakistan from September 2020 to February 2021. Recently delivered mothers and mothers of children till two years of age on breastfeeding/formula feed were consecutively enrolled.

**Result:**

Of 484 participants, breastfeeding was practiced by 180 (37.2%) participants, formula-fed by 85 (17.6%), and mix feed by 219 (45.2%) participants. Out of 185 mothers who had to breastfeed previous babies, 80.2% of mothers still opted to breastfeed their newborns despite the COVID-19 pandemic. Breastfeeding practices exhibited higher incidence in illiterate mothers (aOR 0.229 95% CI 0.05-0.95, p- 0.042), housewives (aOR 0.35 95% CI 0.13-0.95 p-0.040) and shorter length of stay (aOR 0.290 95% CI 0.15-0.57, p- 0.001) while formula /mixed feeding was found higher in mothers with exposure to formula feeding in previous babies (aOR 17.842, 95% CI 8.33-38.19, p- 0.001) and mothers with pain after delivery (aOR 4.526, 95% CI 2.11-9.71, p-<0.001).

**Conclusion:**

Mothers who had to breastfeed their babies in a previous pregnancy, who were less educated, and housewives with a shorter stay in hospital have shown a stronger association with breastfeeding whereas mothers who had previous exposure to formula milk and pain after delivery have shown association to formula feed or mixed feed.

## INTRODUCTION

World Health Organization recommends that breastfeeding be initiated within the first hour of life, and it should be continued by exclusive breastfeeding, at six months introduction of complementary food should take place. Breastfeeding for two years of life has multiple health benefits for the mother and baby.^1^ According to Pakistan demographic survey 2017-18, exclusive breastfeeding in less than six months of age increased from 38% in 2012-13 to 48 % in 2017-18 similarly breastfeeding increased from 36% to 52 % for children 2-3 years.^2^ It is a very cost-effective way of providing nutrition with an impact on decreasing the mortality of newborns and decreasing the incidence of childhood diseases. Studies have shown multiple long-term benefits of breastfeeding on newborns.^3^

Available Literature on research conducted on Pakistani mothers of different social strata has shown a decreased incidence of early initiation of breastfeeding and continuation of breastfeeding.^4^-^6^ The education of the community regarding breastfeeding needs to be improved to reduce the high infant mortality rates.

The COVID-19 pandemic has a significant impact on all aspects of life including decreased incidence of breastfeeding due to lack of antenatal services, decreased counseling, inadequate information regarding the spread of disease, and fear of transmission of COVID-19 leading to separation of babies from mothers.^7^ The already decreased prevalence of breastfeeding in our population may thus be adversely affected.

Our study aimed to assess the effects of the COVID-19 pandemic on breastfeeding practices, mothers’ choices, problems faced by mothers, and other factors that affected infant feeding. The rationale was to simultaneously educate the mothers and resolve their queries and apprehensions to improve maternal and neonatal health. A better understanding will help formulate guidelines and policies to assist in mother and newborn care.

## METHODS

It was a cross-sectional dual center descriptive study conducted at the Pediatric and Gynaecology & Obstetric department at PAF Faisal base and Darul Sehat Hospital after getting the approval from Institutional Ethical Board Review. The study period was from September 2020 to February 2021. A questionnaire and interview techniques were employed to collect data from nursing mothers. Considering the estimated prevalence of breastfeeding in Pakistan as 38%, with a 95 % confidence interval and 4.4% margin of error we calculated the minimum sample size of 457 using the appropriate formula. An estimated sample of 484 patients was finally decided for this study.

### Study population:

The study was conducted in two tertiary care hospitals in Pakistan. The study population consisted of women with children up to two years of age, who visited the Pediatric and OBGYN outpatient departments of both the hospitals.

### Inclusion criteria:

Recently delivered mothers and mothers of children till two years of age on breastfeed/formula feed were included in the study.

### Exclusion criteria:

Mothers whose babies were sick, preterm, or admitted to Neonatal intensive care unit, mothers with Nipple abnormality, with active and Multidrug-resistant Tuberculosis, on chemotherapy or radioactive drugs, mentally unstable with psychiatric issues, and having Babies with any congenital defects like congenital heart disease and cleft lip/palate were excluded from the study.

The ethical approval was taken from the ethical review board committee of the Fazaia Ruth Pfau Medical College (FRPMC) reference number (IRB/07) dated August 4, 2020.

### Data collection:

The respondents were selected by consecutive sampling and an interview questionnaire was filled by the trained junior doctors interviewing the patient. Mothers visiting the Pediatric & Gynae & Obstetrics outpatient department at the tertiary care centers were interviewed after the informed consent.

The questionnaire included demographic details like (mother’s age, education, parity, and occupation), previous feeding practices, current feeding practices, any family member suffering from COVID-19, reasons for not breastfeeding, perception of mothers regarding feeding practices during COVID-19, complications or pain after delivery, lactation counseling advice received during the stay in hospital and length of stay in hospital.

### Data analysis:

SPSS version 24 was used for statistical analysis. The frequencies and percentages were calculated for all qualitative/categorical variables. A Chi-square test was applied to compare the current feeding practice with baseline characteristics, pregnancy, and delivery-related variables. The p-value of ≤0.05 was considered significant. Multinomial logistic regression was also applied to assess the factors associated with current breastfeeding practices. All variables found significant in the contingency table were selected for regression analysis. Breastfeeding was considered the reference category.

## RESULTS

The majority of our participants 222 (45.8%) were 26-30 years of age. Normal vaginal delivery was observed in 213 (44%) participants. Parity status showed that multiparity was predominantly higher among participants, i.e., 391 (80.8%). The length of hospital stay was one day in 138 (28.5). More than half of the participants were graduate or postgraduate, i.e., 252 (52.1%). Only two mothers out of 484 suffered from COVID-19.

Current feeding practice showed that breastfeeding was observed in 180 (37.2%) participants, formula feeding in 85 (17.6%), whereas mixed feeding in 219 (45.2%) participants. A significant association of current feeding practice was observed with education level (p-value 0.016), occupation (p-value 0.002), parity (p-value <0.001), and previous feeding practice (p-value <0.001) as shown in Table-I.

**Table-I:** Comparison of current feeding practice with baseline characteristics (n=484)

Variables	Current feeding practice				p-value

	Breastfeed (n=180)	Formula feed (n=85)	Mix Feed (n=219)		

	N	n (%)	n (%)	n (%)	
***Age, years***					
<25	100	36 (20.0)	18 (21.2)	46 (21.0)	0.999
26-30	222	84 (46.7)	37 (43.5)	101 (46.1)	
31-35	117	44 (24.4)	21 (24.7)	52 (23.7)	
36 & above	45	16 (8.9)	9 (10.6)	20 (9.1)	
***Education level***					
Illiterate	52	29 (16.1)	3 (3.5)	20 (9.1)	0.016
Intermediate or less	180	65 (36.1)	38 (44.7)	77 (35.2)	
Graduate or more	252	86 (47.8)	44 (51.8)	122 (55.7)	
***Occupation***					
Housewife	423	165 (91.7)	64 (75.3)	194 (88.6)	0.002
Employed	61	15 (8.3)	21 (24.7)	25 (11.4)	
***Parity***					
Primiparous	71	13 (7.2)	10 (11.8)	48 (21.9)	<0.001
Multiparous	391	159 (88.3)	72 (84.7)	160 (73.1)	
Grand Multiparous	22	8 (4.4)	3 (3.5)	11 (5.0)	
***Previous feeding (n=413)***					
Breastfeed	185	134 (80.2)	16 (21.3)	35 (20.5)	<0.001
Formula feed	51	1 (0.6)	33 (44.0)	17 (9.9)	
Mix Feed	177	32 (19.2)	26 (34.7)	119 (69.6)	

Similarly, length of hospital stays (p-value <0.001), complications in delivery (p-value 0.042), pain after delivery (p-value <0.001), and immediate family members suffering from COVID-19 (p-value 0.029) were also found to be significantly associated with current feeding practice. (Table-II).

**Table-II:** Comparison of current feeding practice with pregnancy and delivery related variables (n=484).

Variables	Current feeding practice				p-value

	Breastfeed	Formula feed	Mix Feed		

	N	n (%)	n (%)	n (%)	
***Length of hospital stay***					
1 day	138	82 (45.6)	13 (15.3)	43 (19.6)	<0.001
2 days	142	43 (23.9)	25 (29.4)	74 (33.8)	
More than 2 days	204	55 (30.6)	47 (55.3)	102 (46.6)	
Mode of delivery					
NVD*	213	78 (43.3)	43 (50.6)	92 (42.0)	0.390
LSCS/VVD**	271	102 (56.7)	42 (49.4)	127 (58.0)	
***Complications in delivery***					
Yes	64	18 (10.0)	18 (21.2)	28 (12.8)	0.042
No	420	162 (90.0)	67 (78.8)	191 (87.2)	
***Pain after delivery***					
Yes	216	110 (61.1)	30 (35.3)	128 (58.4)	<0.001
No	268	70 (38.9)	55 (64.7)	91 (41.6)	
***Immediate Family Member Suffering from Covid 19***					
Yes	54	12 (6.7)	9 (10.6)	33 (15.1)	0.029
No	430	168 (93.3)	76 (89.4)	186 (84.9)	
***Lactation Counseling***					
Yes	252	101(56.1)	41(48.2)	110(50.2)	0.372
No	232	79(43.9)	44(51.8)	109(49.8)	

* NVD: Normal vaginal delivery,

** LSCS: Lower segment cesarean section, **VVD: Vacuum vaginal delivery.

Illiterate women were 78% less likely to practice formula feeding than breastfeeding (aOR 0.229 95% CI 0.05-0.95, p-value 0.042). Women who practiced formula feed in previous children (were 17.84 times more likely to continue practicing formula/mixed feed than breastfed (aOR 17.842, 95% CI 8.33-38.19, p-value 0.001). Women with one day of the length of hospital stay were 78% less likely to practice formula feeding than breastfeeding (aOR 0.227 95% CI 0.01-0.53, p-value 0.001). Women who reported pain after delivery were 4.52 times more likely to practice formula feeding than breastfeeding (aOR 4.526, 95% CI 2.11-9.71, p-value <0.001). Furthermore, women who were practicing formula/mixed feed before were 20.64 times more like to continue practicing mixed feed than breastfeeding (aOR 20.64, 95% CI 11.28-37.77, p-value 0.001). Women with one day of the length of hospital stay were 71% less likely to practice mixed feed than breastfed (aOR 0.290 95% CI 0.15-0.57, p-value 0.001) (Table-III).

**Table-III:** Multinomial Regression analysis of the variables associated with breastfeeding (n=484).

	Univariate analysis				Multivariate analysis			

	Formula feed		Mixed Feed		Formula feed		Mixed Feed	

	OR (95% CI)	p-value	OR (95% CI)	p-value	aOR (95% CI)	p-value	aOR (95% CI)	p-value
***Education level***								
Illiterate	0.20 (0.05-0.70)	0.012	0.48 (0.26-0.92)	0.026	0.22 (0.05-0.95)	0.042	0.54 (0.21-1.41)	0.208
Intermediate or less	1.14 (0.67-1.96)	0.629	0.83 (0.54-1.28)	0.412	1.24 (0.58-2.65)	1.242	0.56 (0.29-1.07)	0.077
Graduate or more	1		1		1		1	
***Occupation***								
Housewife	0.27 (0.13-0.57)	<0.001	0.70 (0.36-1.38)	0.310	0.35 (0.13-0.95)	0.040	1.04 (0.41-2.64)	0.933
Employed	1		1		1		1	
***Parity***								
Primiparous	1.71(0.72-4.08)	0.224	3.61 (1.89-6.89)	<0.001	1.18 (0.21-6.69)	0.845	0.63 (0.17-2.40)	0.507
Multi/Grand Multiparous	1		1		1		1	
Previous feeding (n=413)*								
Formula/mixed feed	14.97 (7.65-29.29)	<0.001	15.78 (7.65-29.29)	<0.001	17.84 (8.33-38.19)	0.001	20.64 (11.28-37.77)	<0.001
Breastfeed	1		1		1		1	
***Length of stay***								
1 day	0.18(0.09-0.68)	<0.001	0.28 (0.17-0.46)	<0.001	0.22 (0.09-0.53)	0.001	0.29 (0.15-0.57)	<0.001
2 days	0.68 (0.36-1.27)	0.229	0.92 (0.56-1.53)	0.769	0.98 (0.43-2.26)	0.974	1.20 (0.61-2.38)	0.596
More than 2 days	1		1		1		1	
***Complications in delivery***								
Yes	2.41 (1.19-4.93)	0.015	1.31 (0.70-2.47)	0.387	0.99 (0.37-2.65)	0.994	1.10 (0.45-2.67)	0.826
No	1		1		1		1	
***Pain after delivery***								
Yes	2.88 (1.68-4.93)	<0.001	1.11 (0.75-1.67)	0.589	4.52 (2.11-9.71)	<0.001	1.26 (0.67-2.37)	0.469
No	1		1		1		1	
***Immediate Family Member Suffering from Covid 19***								
Yes	1.65 (0.67-4.10)	0.274	2.48 (1.24-4.97)	0.010	1.61 (0.50-5.17)	0.422	2.33 (0.92-5.95)	0.075
No	1		1		1		1	

* n: 413 as 71 mothers out of 484 were primigravida with no previous feeding practice.

**Fig.1:**
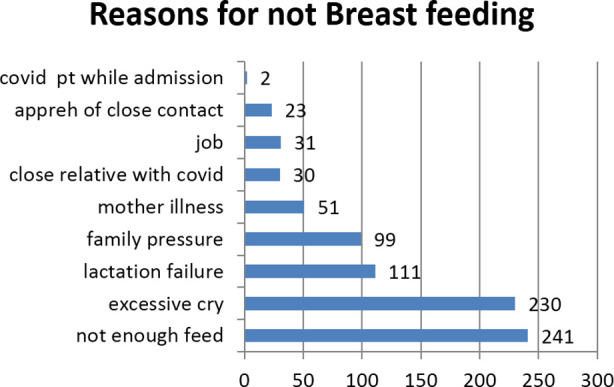
Reasons for not breastfeeding.

Of the 304 women practicing formula or mixed feeding, the most common reason for not breastfeeding as reported by the participants was not enough to feed soon after delivery 241 (79.3%) (Fig.1).

**Fig.2:**
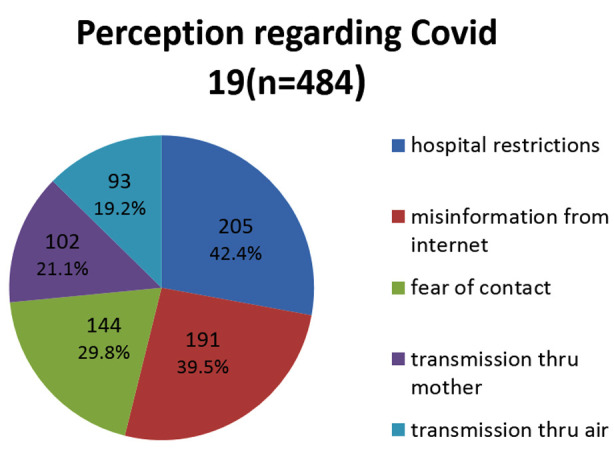
Perceptions of mothers regarding feeding practices during COVID-19

Hospital restrictions for family members during the pandemic leaving mothers alone with the baby in the ward was the most common negative factor affecting breastfeeding as reported by 205 (42.4%) women (Fig.2).

## DISCUSSION

Breastfeeding has a beneficial effect on the survival, overall health, growth, and development of a child. The benefits and recommendations for breastfeeding have been acknowledged and accepted globally.^8^ During the COVID-19 Pandemic particularly the first wave, varied types of recommendations were put forward confusion, thus breastfeeding practices have were affected worldwide.^9^,^10^

In our study, 37.2 % of mothers opted to breastfeed their babies while 45.2 % preferred mixed feeding. This finding is similar to the data given by the demographic survey of Pakistan 2019-2020.^2^ When compared to developed countries, a similar finding was revealed in a study done in Italy where 31.2 % of mothers breastfed at 90 days and 54.3 % at 30 days of lockdown due to COVID-19(8). While a study done in the UK revealed that 58.6 % of mothers were breastfeeding and 22.5% preferred mix feeding.^11^

Comparing current breastfeeding practices with previous feeding practices in our mothers, the majority of them who breastfed their previous children choose to breastfeed this time despite the COVID-19 Pandemic. While the mothers who preferred to mix feed in previous feeding practices, 19.2 % of those choose to breastfeed this time during the COVID-19 Pandemic.

This fact is supported by the study in Belgium, where 91% of mothers continued the same plan of feeding despite the COVID-19 pandemic, and 86% of the mother who had previously breastfed their babies did not alter the method of feeding while 82% of mothers extended the frequency of feeding being at home due to lockdown.^12^ A survey done by other researchers revealed that 2.6 % of mothers changed their method from formula feed to breastfeeding owing to the unavailability and cost of formula milk, apprehension of contaminated products, and feeling of breast milk is safe for their infants.^13^

This explains that although the Covid-19 pandemic has affected multiple aspects of our life but did not affect breastfeeding practices by a large number of mothers.^11^ Mother’s belief toward breastfeeding and the protection it confers to their babies is more sustainable and strong than the impact of the COVID-19 pandemic. Even in a lockdown situation, breastfeeding was the secure and safest way to protect the babies as seen in countries like the United Kingdom where the majority of mothers attributed breastfeeding was impregnable during lockdown.^11^

As more than half of our participants were graduates, most opted for mixed feeding. Employed mothers who were working from home had to take on the additional task of child care as well as home responsibilities which made them go for mixed feeding. Lack of family support and pressure from family members has been an additional cause of initiating formula feed. This has been observed in a study in the United Kingdom that poor emotional and social support is an important factor in reinforcing the impact of the COVID-19 pandemic on breastfeeding.^14^

Mothers who were housewives were used to breastfeeding and they continued to do so during a pandemic. Most partners working from home have given them a helping hand and hence more time to breastfeed. The study in Bangladesh has a similar opinion that women with joint families had a lot of support from their In-laws as well as their partner during the pandemic.^15^

Mothers who have given birth to their first baby during a pandemic are the ones who were affected the most. The majority of them opted for mixed feeding in our study. Reasons could be pressure and stress of being alone in the hospital as most relatives and visitors were not allowed, fewer chances for lactation counseling during the pandemic, apprehension of transmission of the virus from milk, transmission through contact with staff and health professionals, and no previous experience of breastfeeding. These were supported by studies that confirmed that mothers got less support who delivered during the pandemic as compared to mothers who gave birth before the pandemic, additionally, 13.2% of mothers were more concerned about the safety of breastfeeding during the pandemic.^11^,^16^ New mothers apprehend of breastfeeding being safe and protected was well explained by Kronborg and Hall in their study.^17^

In our study, it has been observed that the mothers who spent less time in hospital after delivery were more likely to go breastfeeding. This is contrary to a study done by Alison Stuebe, which says shorter stay in hospital gives lesser support to mothers.^18^ During the pandemic, they feel more relaxed and content being at home with other family members who can help them. Being comfortable favors them to breastfeed and hence fewer chances of acquiring formula feed.^15^ On the other hand, mothers who had pain during or after delivery are less likely to adapt to breastfeeding as being alone and difficult to handle the baby all by themselves. By switching to formula or mixed feed, they can get ample time to rest.

In our study mothers who had relatives suffering from covid at home chose to keep their babies on mixed feed because of apprehension of transferring the virus to their babies as being exposed to surrounding with Covid patient at-home quarantine. Many mothers had to face an increase in workload because of the absence of domestic workers and unavailability of other relatives for help or relatives avoided visitation due to pandemic lockdown. Similar findings were shared by studies.^15^,^19^ Mothers had to follow the instruction during pandemics about cleaning, hygiene, and social distancing which were an additional burden to bear and made mothers stressed out, leading to a decrease in breastfeeding or going towards formula feed.

Many factors contribute to the initiation, continuation, and cessation of breastfeeding. Support from family and health professionals is of prime importance during the stay at the hospital and after discharge, as this contributes to positive and negative impacts on the mother’s attitude towards breastfeeding.

In our study, there were few experiences shared by our patients, they felt alone in the ward due to covid-19 restrictions for family members, they had misinformation from the internet and family members had fear of contact virus from doctors/ nurses during breastfeeding and from the surrounding. Similar apprehension was also shown in a study conducted by Mortazavi et al.^20^

Our study will help the policymakers in formulating guidelines for parents and health care professionals regarding the importance of breastfeeding practices during the COVID-19 Pandemic and support to mothers while in hospital.

### Limitations

It couldn’t assess the socioeconomic status of these mothers. Although it represents all strata of a woman coming to the Outpatient department from different localities, still our analysis cannot be a representation of all mothers in Pakistan.

## CONCLUSION

A significant association between breastfeeding was observed with education level, occupation, parity, previous feeding practice, and length of hospital stay. Housewives, illiterate mothers, and mothers with a shorter stay in hospital were 65 to 75 times less likely to practice formula feeding than breastfeeding. Whereas mothers who had pain after delivery and mothers with exposure to formula feed in previous babies were 4.5 to 17 times more likely to practice formula feeding than breastfeeding respectively, Mothers with family members suffering from COVID-19 and primigravida were also found to be associated with formula /mixed feed.

### Author’s contribution:

SQB, SAM, KMAK: Conception and design:.

SQB, UHAS: Data collection:

SQB. Analysis and Interpretation of result:

SQB, SAM, UHAS: Draft Manuscript Preparation:

KMAK, SQB: Final Review:

SQB: Accuracy and integrity of the work:
